# Epac1 interacts with importin β1 and controls neurite outgrowth independently of cAMP and Rap1

**DOI:** 10.1038/srep36370

**Published:** 2016-11-03

**Authors:** Faiza Baameur, Pooja Singhmar, Yong Zhou, John F. Hancock, Xiaodong Cheng, Cobi J. Heijnen, Annemieke Kavelaars

**Affiliations:** 1 Laboratory of Neuroimmunology, Department of Symptom Research, Division of Internal Medicine, The University of Texas MD Anderson Cancer Center, Houston, TX 77030, USA; 2 Department of Integrative Biology and Pharmacology and Texas Therapeutics Institute, The University of Texas Health Science Center, Houston, TX 77030, USA

## Abstract

Exchange protein directly activated by cAMP-1 (Epac1) is a cAMP sensor that regulates multiple cellular functions including cellular migration, proliferation and differentiation. Classically, Epac1 is thought to exert its effects through binding of cAMP leading to a conformational change in Epac1 and its accumulation at the plasma membrane (PM) where it activates Rap1. In search for regulators of Epac1 activity, we show here that importin β1 (impβ1) is an Epac1 binding partner that prevents PM accumulation of Epac1. We demonstrate that in the absence of impβ1, endogenous as well as overexpressed Epac1 accumulate at the PM. Moreover, agonist-induced PM translocation of Epac1 leads to dissociation of Epac1 from impβ1. Localization of Epac1 at the PM in the absence of impβ1, requires residue R82 in its DEP domain. Notably, the PM accumulation of Epac1 in the absence of impβ1 does not require binding of cAMP to Epac1 and does not result in Rap1 activation. Functionally, PM accumulation of Epac1, an Epac1 mutant deficient in cAMP binding, or an Epac1 mutant tethered to the PM, is sufficient to inhibit neurite outgrowth. In conclusion, we uncover a cAMP-independent function of Epac1 at the PM and demonstrate that impβ1 controls subcellular localization of Epac1.

Exchange protein directly activated by cAMP (Epac) 1 and 2 are sensors for the universal second messenger cAMP. They function as guanine exchange factors for small G proteins of the Rap and Ras family^1^,^2^. Epac1 is ubiquitously expressed and contributes to various pathologies, including cardiac hypertrophy, myocardial infarction, Alzheimer’s disease, chronic obstructive pulmonary disease, inflammation, diabetes, obesity, cancer, and pain^3^,^4^,^5^,^6^,^7^,^8^,^9^,^10^,^11^,^12^,^13^,^14^,^15^. Epac1 regulates a plethora of cellular processes such as differentiation, proliferation, cell adhesion, and actin dynamics to name a few^16^,^17^,^18^,^19^,^20^,^21^,^22^,^23^. In the nervous system, Epac1 has been implicated in the regulation of axon guidance and elongation as well as in neurite outgrowth^16^,^17^,^24^,^25^,^26^.

In the absence of cAMP, Epac proteins assume an auto-inhibited conformation in which the catalytic site is covered by the regulatory domain^27^. Crystal structure analysis of Epac2 demonstrates that binding of cAMP to Epac relieves the protein from its auto-inhibitory conformation leading to Rap1 activation and downstream signaling^28^,^29^. Overexpression studies have shown that under baseline conditions Epac1 resides at the nuclear envelope and is also present in the cytosol in multiple different cell lines^30^,^31^,^32^,^33^. Increases in cellular cAMP promote translocation of Epac1 to the plasma membrane (PM), thus allowing localized Rap1 activation^31^. This cAMP-induced translocation of Epac1 to the PM is thought to depend on passive diffusion and requires residue R82 in the Dishevelled, Egl-10, and Pleckstrin (DEP) domain to bind phosphatidic acid (PA) at the PM. The current model is that the cAMP-induced conformational change in Epac1 increases solvent exposure of this region in the DEP domain to promote binding of Epac1 to PA at the PM^31^,^34^.

Multiple proteins contribute to the regulation of the subcellular localization of Epac1. For example, the A-kinase anchoring protein mAKAP^35^,^36^, RanBP2 and RAN have been implicated in the perinuclear localization of Epac1^37^,^38^. The interaction of Epac1 with RanBP2 has been shown to regulate local Epac1 activity and signaling to Rap1^37^,^38^. In addition, Epac1 interacts with microtubules and AKAP9 and this interaction is involved in regulation of microtubule elongation and endothelial barrier properties^39^. Recently, we demonstrated that phosphorylation of Epac1 by the kinase GRK2 inhibits agonist-induced PM accumulation of Epac1 and Rap1 activation, thereby preventing chronic pain^7^,^40^,^41^. The aim of the present study was to get more insight in the regulation of Epac1 subcellular localization and function. We used proteomics to identify Epac1-binding proteins. The results show that importin β1 (impβ1) is an Epac1 binding partner that regulates Epac1 subcellular localization. Furthermore, we uncovered a thus far unidentified cAMP-independent function of Epac1 controlled by impβ1 in the regulation of neurite outgrowth.

## Results

### Epac1 interacts with importin β1

**Figure 1:**
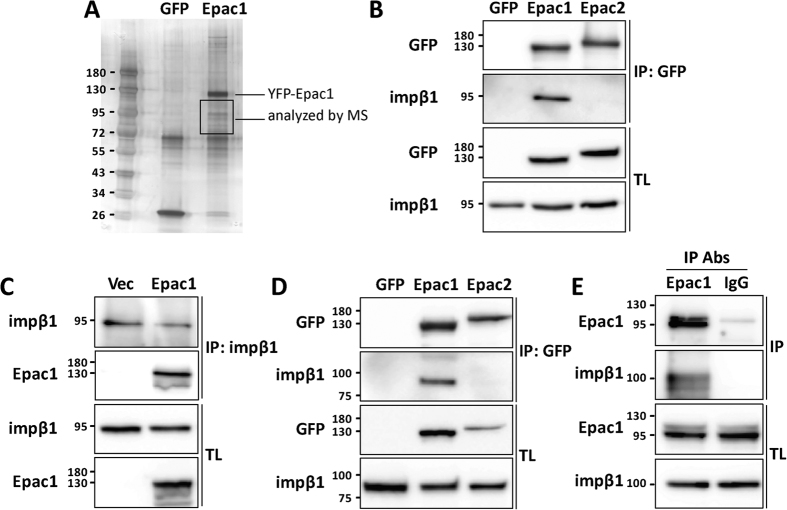
Identification of impβ1 as an Epac1 binding partner. **(A)** Lysates from HEK293 cells overexpressing YFP-Epac1 or GFP were subjected to GFP-TRAP immunoprecipitation (IP) followed by silver staining and mass spectrometry (MS) analyses of bands between 72 and 120 kDa. Mass spectrometry results are presented in Table 1. (**B**) GFP-TRAP precipitates from HEK293 cells expressing YFP-Epac1, YFP-Epac2, or GFP as a negative control, were analyzed by western blotting with impβ1 and GFP antibodies. Levels of impβ1, Epac1, and Epac2 in the IP and total lysate (TL) are shown in representative western blots. **(C)** Impβ1 was immunoprecipitated from cells expressing HA-Epac1 or control vector using impβ1 antibody coupled to protein G-sepharose. Samples were analyzed by western blotting with impβ1 and Epac1 antibodies. Representative western blots are shown. **(D)** Similar to (B), GFP-TRAP precipitates from N2A cells expressing YFP-Epac1, YFP-Epac2, or GFP as a negative control, were analyzed by western blotting. **(E)** Endogenous Epac1 was immunoprecipitated from EA.hy926 cells using Epac1 antibody (A-5) coupled to protein A-sepharose. Proteins were visualized by western blotting using Epac1 and impβ1 antibodies.

In search for novel endogenous regulators of Epac1, we identified Epac1 binding partners using immunoprecipitation followed by mass spectrometry. YFP-Epac1 or control GFP were immunoprecipitated from human embryonic kidney-293 (HEK) cells using GFP-TRAP beads. SDS-PAGE followed by silver staining revealed enrichment of bands migrating at 72–120 kDa in the Epac1-YFP precipitate (Fig. 1A). These bands were analyzed by mass spectrometry. We identified several potential Epac1 binding partners, including impβ1, RanGAP, HSP90A and B, and HSP70 (Table 1). The interaction of RanGAP with Epac1 has recently been characterized^37^,^38^ and the presence of RanGAP in our sample confirms the validity of our approach. HSP90 and HSP70 are members of the heat shock protein family that function as chaperone proteins and interact with a large array of proteins; therefore these hits were not further studied.

One earlier study reported the presence of impβ1 in an Epac1-containing protein complex, but the interaction of these two proteins was not further defined^37^. We pursued this hit by western blotting and *in vivo* co-localization analysis. Impβ1 was detected in the GFP-immunoprecipitate of HEK293 cells overexpressing YFP-Epac1, but not of cells expressing YFP-Epac2 or the control vector GFP (Fig. 1B). To further confirm the interaction between Epac1 and impβ1, we immunoprecipitated impβ1. Epac1 co-immunoprecipitated with impβ1 (Fig. 1C). The interaction between Epac1 and impβ1 was not cell-specific as it was also detected in neuroblastoma 2A (N2A) cells (Fig. 1D). Moreover, we also detected the interaction between endogenous Epac1 and impβ1 in the endothelial cell line EA.Hy926 which expresses high levels of Epac1. As is shown in Fig. 1E, when Epac1 was immunoprecipitated from EA.hy926 cells, impβ1 was detected in the immunoprecipitate (Fig. 1E). These findings identify impβ1 as a specific Epac1 binding partner.

### Importin β1 regulates the subcellular distribution of Epac1

**Figure 2:**
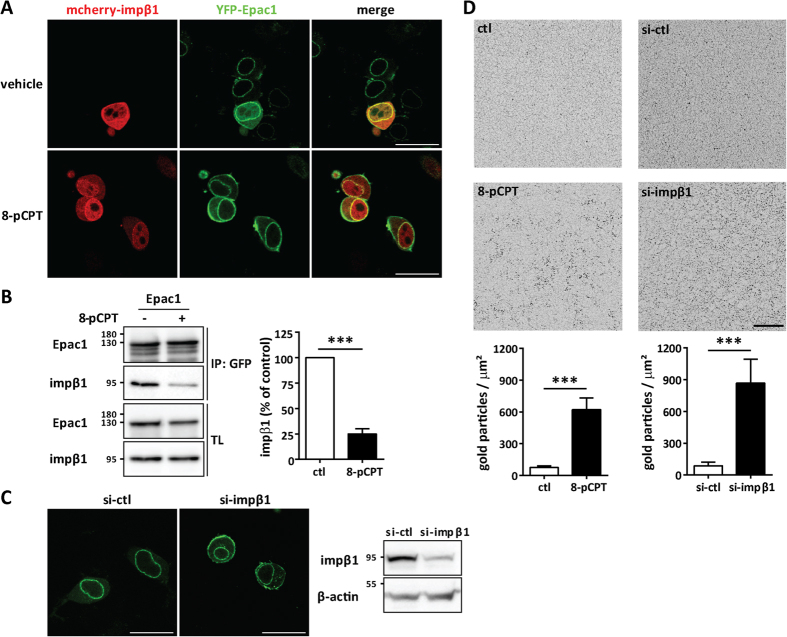
Impβ1 serves as a cytosolic anchor for Epac1. **(A)** N2A cells co-expressing YFP-Epac1 (green) and mcherry-impβ1 (red) were treated with either vehicle or 1 μM 8-pCPT-AM for 15 min, followed by live cell imaging. Representative images of >50 cells in each condition are shown; scale bar corresponds to 25 μm. **(B)** N2A cells expressing YFP-Epac1 or GFP were treated with either vehicle or 10 μM 8-pCPT-AM for 15 min. Epac1 was pulled down using the GFP-TRAP beads and samples were analyzed by western blotting with impβ1 and Epac1 antibodies. **(C)** N2A cells overexpressing YFP-Epac1 were treated with impβ1 si-RNA (si-impβ1) or control si-RNA (si-ctl) and the effect on Epac1 localization was monitored by live cell imaging. Images are representative of at least three independent experiments with >50 cells each; scale bar corresponds to 25 μm. Western blot shows confirmation of impβ1 knockdown. **(D)** PM sheets of control (ctl) EA.hy926 cells or EA.hy926 cells treated with 1 μM 8-pCPT-AM for 15 min (left panels), or EA.hy926 cells treated with si-impβ1 or si-ctl (right panels) were attached to EM grids, immunolabeled with gold nanoparticles coupled to Epac1 antibody (H-70), and imaged by EM. Images are representative of at least 15 PM sheets per condition; scale bar corresponds to 200 nm. Quantification of the number of gold-coupled Epac1 nanoparticles per μm^2^ area in the inner leaflet of the plasma membrane is shown in the bar graphs. Data were analyzed by Student’s t-test; ***P < *0.001*.

We analyzed the subcellular distribution of Epac1 and impβ1 in N2A cells co-expressing YFP-Epac1 and mcherry-impβ1 by live cell fluorescence imaging. Impβ1 is best known for its role in nuclear protein import, but it also plays a role in axonal transport^42^. The results in Fig. 2 show that impβ1 was detected in both cytosolic and nuclear compartments. In line with previous studies, Epac1 localized to the nuclear envelope and cytosol^30^,^31^,^43^. The results in Fig. 2A show that Epac1 and impβ1 co-localized mainly at the nuclear envelope. We also observed co-localization in the cytosol.

Stimulation of Epac1 with the selective Epac agonist 8-pCPT induced its translocation to the PM, but Epac1 activation and redistribution did not affect the localization of impβ1 (Fig. 2A). Stimulation with 8-pCPT reduced the interaction between impβ1 and Epac1 as quantified by immunoprecipitation analysis (Fig. 2B).

To determine whether impβ1 is required to prevent PM accumulation of Epac1 under baseline conditions, we reduced cellular impβ1 levels using si-RNA. Knockdown of impβ1 (si-impβ1) was sufficient to induce Epac1 accumulation at the PM (Fig. 2C). In the control samples (si-ctl) Epac1 localizes to the cytosol and nuclear envelope, as expected (Fig. 2C).

The results in Fig. 2D show that in cells only expressing endogenous Epac1, knockdown of impβ1 (si-impβ1) was also sufficient to induce accumulation at the PM. In these experiments, intact 2-D sheets of PM were prepared^44^,^45^, and the inner PM leaflet immunolabeled with gold nanoparticles coupled directly to an Epac1 antibody. The extent of anti-Epac immunogold labeling was determined by electron microscopy (EM). The results show that decreasing endogenous impβ1 markedly increased the amount of endogenous Epac1 at the PM (Fig. 2D). 8-pCPT-induced accumulation of endogenous Epac1 at the PM was used as a positive control. These results clearly indicate that impβ1 serves as an anchor for Epac1 which prevents Epac1 from accumulating at the PM in the absence of cAMP.

### Accumulation of Epac1 at the PM is sufficient and required to inhibit neurite outgrowth

**Figure 3:**
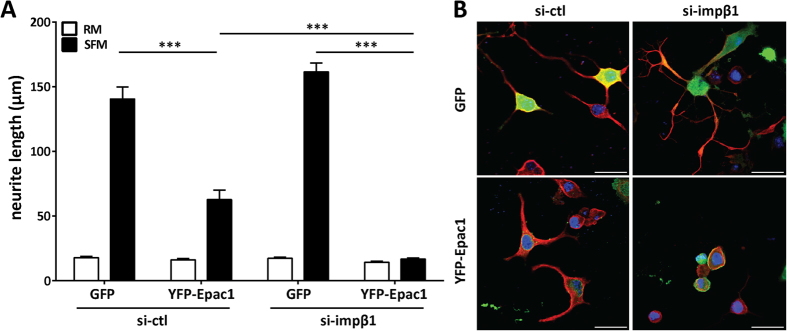
Epac1 inhibits neurite outgrowth, which is exacerbated in the absence of impβ1. **(A)** N2A cells overexpressing YFP-Epac1 or GFP, were cultured in serum-free medium (SFM; solid bars) to induce differentiation or kept in regular growth medium (RM; open bars). Cells were also treated with impβ1 si-RNA (si-impβ1) or control si-RNA (si-ctl). Neurite length was analyzed after 24 hrs as described in the methods section. Data shown are the mean ± SEM neurite lengths for three independent experiments where at least 4 images of >40 cells each were analyzed for each condition. Data were analyzed by Two-Way ANOVA; ***P < *0.0001*. **(B)** Representative images of cells from each condition cultured in serum free medium; YFP-Epac1 (green), βIII-tubulin (red), and DAPI (blue); scale bar corresponds to 25 μm.

Epac1 regulates growth of neurites and axons in hippocampal and dorsal root ganglion neurons^17^,^24^,^25^,^26^. In addition, there is evidence that impβ1 is transcribed in axons of damaged dorsal root ganglion neurons and contributes to their recovery^46^. We therefore examined whether the change in subcellular localization of Epac1 after knockdown of impβ1 affects neurite outgrowth. Neurite outgrowth was induced by culturing N2A cells in serum-free medium (SFM) and neurite length was quantified after 24 hrs. Interestingly, only in cells expressing Epac1, knockdown of impβ1 completely abolished neurite outgrowth. Knockdown of impβ1 did not affect neurite outgrowth in cells expressing the control GFP vector (Fig. 3A,B), and endogenous Epac1 is not detectable in these cells. In cells with normal levels of impβ1 (si-ctl), overexpression of Epac1 partially inhibited neurite outgrowth (Fig. 3).

**Figure 4:**
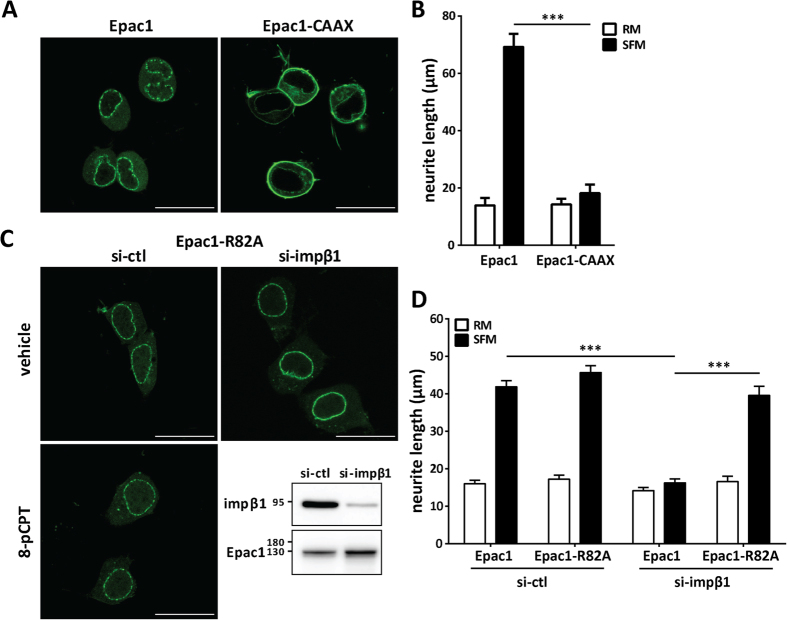
Localization of Epac1 at the plasma membrane is sufficient and required to inhibit neurite outgrowth. **(A)** Representative images of N2A cells expressing Epac1-CAAX to tether Epac1 to the PM or control Epac1; scale bar corresponds to 25 μm. **(B)** N2A cells expressing Epac1 or Epac1-CAAX were grown in either SFM (solid bars) or RM (open bars) for 24 hrs. Data shown are the mean ± SEM neurite lengths for three independent experiments where at least 4 images of >40 cells each were analyzed for each condition. Comparison between Epac1 (SFM) and Epac1-CAAX (SFM) was performed by Student’s t-test; ***P < *0.0001*. **(C)** Cells expressing YFP-Epac1-R82A mutant (deficient in binding phosphatidic acid at the PM) and treated with si-ctl or si-impβ1, were analyzed by live cell imaging. Representative images show that YFP-Epac1-R82A does not localize to the PM when impβ1 is knocked down; scale bar corresponds to 25 μm. Confirmation of the inability of Epac1-R82A to accumulate at the PM in response to 1 μM 8-pCPT-AM is shown in the lower left image. Western blot confirms impβ1 knockdown. **(D)** Cells expressing either YFP-Epac1 or YFP-Epac1-R82A mutant and treated with either si-ctl or si-impβ1 were analyzed for neurite outgrowth after culture in serum-free medium for 24 hrs. Data analyzes were performed by Two-Way ANOVA; ***P < *0.0001*.

Next, we used an Epac1 construct that is tethered to the PM via a CAAX motif at its C-terminus (Epac1-CAAX)^32^ in cells with endogenous levels of impβ1 (Fig. 4A). Tethering of Epac1 to the PM was sufficient to completely block neurite outgrowth without changes in endogenous impβ1 levels (Fig. 4B). These results indicate that Epac1 localization at the plasma membrane is sufficient to inhibit neurite outgrowth even in cells with normal impβ1 levels.

It has been shown that PM accumulation of Epac1 in response to 8-pCPT requires arginine 82 (R82) in the DEP domain; this residue is required for binding of activated Epac1 to its PM anchor phosphatidic acid^34^. To determine whether the binding of Epac1 to the PM in cells depleted of impβ1 also requires R82, we used the Epac1-R82A mutant. The data in Fig. 4C show that Epac1-R82A failed to accumulate at the PM in cells depleted of impβ1. As expected, 8-pCPT stimulation did not result in Epac1-R82A accumulation at the PM, and under control conditions (si-ctl) the distribution of Epac1-R82A was similar to that of wild type (WT) Epac1 (Fig. 4C). These results demonstrate that residue R82 is required for Epac1 localization to the plasma membrane in cells depleted of impβ1.

Notably, R82-mediated localization of Epac1 to the PM is also required for inhibition of neurite outgrowth; knockdown of impβ1 did not have any effect on neurite outgrowth of cells expressing Epac1-R82A, whereas it completely abolished neurite outgrowth in the presence of WT Epac1 (Fig. 4D). The R82A mutation did not affect the Epac1/impβ1 interaction (Supplementary Figure S1). These data support the hypothesis that Epac1 localization at the PM in the absence of impβ1 is not only sufficient but also required for inhibiting neurite outgrowth.

### Inhibition of neurite outgrowth by Epac1 is not mediated by cAMP signaling

**Figure 5:**
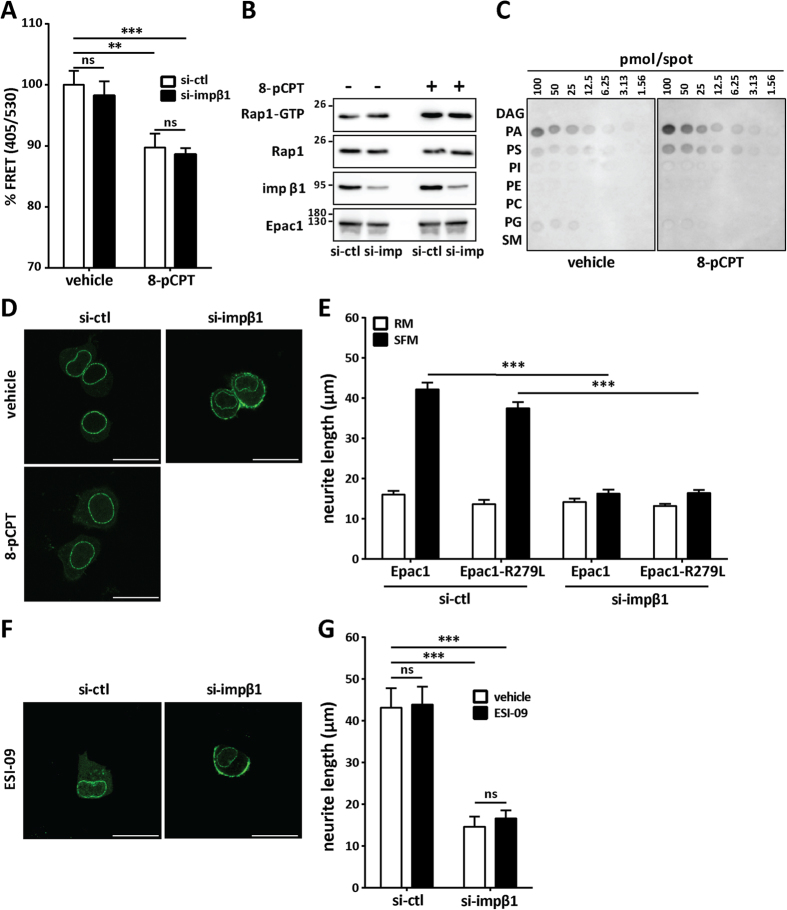
Knockdown of impβ1 promotes membrane accumulation of Epac1 and inhibition of neurite outgrowth independently of cAMP binding. **(A)** N2A cells overexpressing the Epac1 FRET reporter were treated with si-ctl or si-impβ1 and analyzed by flow cytometry following treatment with either vehicle or 30 μM 8-pCPT-AM for 15 min. Bar graph shows the percentage of the FRET ratio (405/530) in YFP-positive cells, in si-ctl (open bars) as compared to si-impβ1 (solid bars) samples. **(B)** N2A cells overexpressing YFP-Epac1 were treated with si-ctl or si-impβ1, followed by treatment with vehicle or 0.1 μM 8-pCPT-AM for 15 min. Rap1-GTP was pulled down with Ral GDS-Rap binding domain beads followed by western blotting to detect Rap1-GTP and total Rap1, impβ1, and Epac1. **(C)** Binding of purified Epac1 to membrane lipid strips in the absence or presence of 50 μM 8-pCPT for 15 min. **(D)** Representative images of N2A cells overexpressing YFP-Epac1-R279L mutant (deficient in binding cAMP) and treated with si-ctl or si-impβ1 were analyzed by live cell imaging. Representative images show that YFP-Epac1-R279L localizes to the PM when impβ1 is knocked down. Lower left image confirms that Epac1-R279L does not respond to stimulation with 8-pCPT-AM. **(E)** N2A cells overexpressing either YFP-Epac1-R279L mutant or YFP-Epac1 and treated with si-ctl or si-impβ1, were grown in SFM (solid bars) or RM (open bars). Neurite length was analyzed as described in the legend to Fig. 3. (**F**) The Epac inhibitor ESI-09 does not prevent the effect of impβ1 depletion on PM accumulation of Epac1. N2A cells overexpressing YFP-Epac1 were treated with si-ctl or si-impβ1 and 5 μM ESI-09. Representative images showing that YFP-Epac1 also localizes to the PM in the presence of ESI-09 when impβ1 is knocked down. **(G)** N2A cells overexpressing YFP-Epac1 treated with si-ctl or si-impβ1, were grown in SFM supplemented with vehicle (open bars) or 5 μM ESI-09 (solid bars) for 24 hrs. Data in all panels were analyzed by Two-Way ANOVA; ns: not significant, ***P < *0.001*, and **P < *0.01*. Scale bar in all images corresponds to 25 μm.

It is well established that cAMP binding induces a conformational change in Epac1 leading to its translocation and Rap1 activation at the PM^27^,^32^,^47^,^48^,^49^. In our experiments, Epac1 accumulates at the PM in cells deficient of impβ1 without addition of an Epac1 agonist. It may be possible, however, that knockdown of impβ1 increases cellular cAMP levels thereby promoting Epac1 PM accumulation. To address this, we first performed fluorescence resonance energy transfer (FRET) analyses using an ECFP-Epac1-citrine FRET probe^32^. As a positive control, we show that treatment of cells with 8-pCPT reduced the FRET signal, indicating a conformational change in Epac1 (Fig. 5A). Importantly, knockdown of impβ1 did not induce a detectable change in the FRET signal, indicating that the decrease in impβ1 did not increase cellular cAMP (Fig. 5A). Furthermore, knocking down impβ1 did not activate Rap1, as we did not detect an increase in GTP-bound Rap1 (Fig. 5B). Additionally, Rap1 activation in response to 0.1 μM 8-pCPT was similar in si-ctl and si-impβ1 cells (Fig. 5B). These findings indicate that Epac1-mediated inhibition of neurite outgrowth is independent of Rap1 activation, and support the hypothesis that accumulation of Epac1 at the PM as well as inhibition of neurite outgrowth in the absence of impβ1, is independent of cAMP-mediated activation of Epac1.

It is known that agonist-activated purified Epac1 binds to PA, but not to other phospholipids^34^. We used an *in vitro* protein/lipid overlay assay to determine whether purified Epac1 binds to PA. The results in Fig. 5C show that even in the absence of agonist, purified Epac1 bound to PA, indicating that a cAMP-induced conformational change in Epac1 is not required to allow PA binding. In line with earlier studies, 8-pCPT increased binding to PA.

To further test the hypothesis that Epac1 accumulation at the PM does not require agonist-induced Epac1 activation, we used both an Epac1 mutant deficient in cAMP-binding Epac1-R279L^31^,^33^ and the Epac1 inhibitor ESI-09^50^. Notably, in cells depleted of impβ1, Epac1-R279L accumulated at the PM like WT Epac1. As expected, Epac1-R279L did not translocate to the PM in response to 8-pCPT, confirming that this mutant does not respond to cAMP (Fig. 5D). Consistently, addition of the Epac inhibitor ESI-09 did not prevent Epac1 accumulation at the PM in cells depleted of impβ1 (Fig. 5F).

The data in Fig. 5E show that both Epac1-R279L and WT Epac completely inhibited neurite outgrowth in the absence of impβ1. In line with a cAMP-independent effect of Epac1 on neurite outgrowth in cells depleted of impβ1, addition of ESI-09 did not affect neurite outgrowth (Fig. 5G). In summary, our results support a model in which PM accumulation of Epac1 strongly inhibits neurite outgrowth via a mechanism that is independent of cAMP-signaling.

## Discussion

Epac proteins are widely expressed and have been implicated in multiple (patho)physiological processes including neuronal differentiation, cardiac function, insulin signaling and diabetes, chronic pain, and airway functioning (reviewed in refs 51 and 52). In search for novel regulators of the subcellular distribution and function of Epac1, we identified impβ1 by mass spectrometry analyses. We also uncover a cAMP-independent function of Epac1 at the plasma membrane in the regulation of neurite outgrowth.

Impβ1 and other members of the nuclear pore complex, including RanBP2 have previously been identified as potential Epac1 binding partners on the basis of a proteomic analyses^37^,^38^. However, the functional importance of the Epac1/impβ1 interaction remained to be elucidated. We show here for the first time that Epac1 and impβ1 co-localize at the nuclear envelope as well as in the cytosol. Moreover, we demonstrate that both endogenous and overexpressed Epac1 accumulate at the PM in cells depleted of impβ1, suggesting that impβ1 binding to Epac1 serves to prevent accumulation of Epac1 at the PM. It is well-known that cAMP binding to Epac1 induces translocation to the PM^31^,^34^,^49^. However, our data indicate that in the absence of impβ1, cAMP binding is not required for the PM accumulation of Epac1. Functionally, it is known that cAMP-mediated activation of Epac1 promotes neurite outgrowth^16^,^17^,^53^. In contrast, we show here that cAMP-independent plasma membrane accumulation of Epac1 inhibits neurite outgrowth. In summary, we uncover a thus far novel cAMP-independent function of Epac1. Moreover, we characterize an unappreciated role for impβ1 in controlling Epac1 subcellular localization and thereby neurite outgrowth.

cAMP induces a conformational change in Epac1 and stimulates its accumulation at the PM. This agonist-induced PM accumulation of Epac1 is dependent on amino acid R82 in the DEP domain of Epac1. It has been proposed that cAMP-induced conformational changes in the DEP domain of Epac1 reorients R82, thereby facilitating binding to PA^34^. In support of this model, *in vitro* studies showed that binding of purified Epac1 to PA is increased in the presence of the Epac agonist 8-pCPT^34^. The data we present in Fig. 5C demonstrate that purified Epac1 binds PA in the absence of agonist stimulation, while binding is increased in response to agonist. In addition, the crystal structure of Epac2 indicates that the residue equivalent to R82 in Epac1 is exposed even in the absence of cAMP, as was noted by Consonni *et al.*^34^. Thus, although cAMP-induced changes in the conformation of Epac1 likely increase the binding to PA, the existing model did not fully explain how PM accumulation of Epac1 in the absence of elevated cAMP is prevented. Our present data provide an explanation. Under basal conditions, Epac1 is bound to impβ1 and localizes to the nuclear envelope and cytosol. We show here that upon stimulation with 8-pCPT, Epac1 and impβ1 dissociate and Epac1 accumulates at the PM. Depleting cells from impβ1 is sufficient for Epac1 to accumulate at the PM. We therefore propose that the conformational change in Epac1 that is induced by cAMP binding is responsible for dissociation from impβ1, and that it is this dissociation that allows the free Epac1 to bind to PA in the PM. In line with our hypothesis, we demonstrate that Epac1 translocates to the PM in the absence of impβ1, and this process does not require binding of cAMP to Epac1. These findings are consistent with our model that Epac1 is prevented from R82-mediated PM association by being in a complex with impβ1.

It should be noted that a major proportion of Epac1 is still detected at the nuclear envelope even in the absence of impβ1 or upon 8-pCPT stimulation. It has been proposed that it is in fact cytosolic, and not nuclear Epac1 that translocates to the PM in response to agonist^31^,^34^. If this is the case it would mean that impβ1 mainly serves as a cytosolic anchor for Epac1, and that other or additional proteins anchor Epac1 at the nuclear envelope. Indeed there is evidence that Epac1 not only interacts with impβ1, but also with the nuclear pore complex proteins RanBP2, nucleoporin 205, nucleoporin 98, and Ran, a protein that binds to nucleoporins^37^,^38^.

Functionally, we propose that cAMP-independent accumulation of Epac1 at the PM is sufficient for inhibition of neurite outgrowth. This hypothesis is supported by five lines of evidence. First, tethering of Epac1 to the PM using a CAAX motif was sufficient to abolish neurite outgrowth without changing impβ1 levels. Second, in cells lacking impβ1, an Epac1 mutant that is not capable of binding cAMP accumulates at the PM and inhibits neurite outgrowth as effectively as WT Epac1. Third, the Epac inhibitor ESI-09 did not prevent PM accumulation of Epac1 in cells depleted of impβ1 or the associated inhibition of neurite outgrowth. Fourth, depletion of impβ1 did not change Epac1 conformation as detected using an Epac1 FRET probe or induce Rap1 activation, indicating that Epac1 was not activated by cAMP. Fifth, while it is known that cAMP binding to Epac1 induces DNA-PK export from the nucleus^54^, redistribution of Epac1 in cells depleted of impβ1 does not affect nuclear export of DNA-PK in response to cAMP (Supplementary Figure S2). This finding indicates that there is no change in this function of Epac1 at the nuclear envelope that could explain our results. However, we cannot exclude that other potential changes in Epac1 activity at the nuclear envelope contribute to the observed changes in neurite outgrowth.

It has long been known that neuronal differentiation, and in particular formation of the axon and dendrites, is controlled by cAMP signaling^24^,^25^,^55^,^56^,^57^. Initially, this was thought to be mediated exclusively by PKA, but it is now known that there is also a prominent role for cAMP signaling to Epac1 in promoting neurite outgrowth^25^. For example, it was shown recently that Epac1 depletion reduced polarization of hippocampal neurons *in vitro* and produced shorter axons. The role of Epac1 in neuronal polarization was ascribed to activation of Rap1b downstream of activation of Epac1 by cAMP, and Rap1b activation is necessary for establishment of polarity in hippocampal neurons^26^. Thus, the role of Epac1 in promoting axon-formation is thought to occur downstream of cAMP binding to Epac1 leading to Rap1 activation. Interestingly, and in line with our current observations, Munoz-Llancao *et al.* reported data showing that overexpression of an Epac1 mutant that does not bind cAMP, and that the authors refer to as a dominant negative mutant, reduces axon length. In the light of our present findings, it may well be that the inhibitory effect of the Epac1 mutant that does not bind cAMP is in fact reflecting Epac1-mediated inhibition of neurite outgrowth in the absence of agonist binding to Epac1.

We can only speculate on the mechanisms underlying the observed effect of cAMP-independent PM accumulation of Epac1 on neurite outgrowth. One possibility is Epac1-mediated regulation of microtubule dynamics. Epac1 interacts directly with microtubules and *in vitro* promotes microtubule formation even in the absence of cAMP^39^,^58^,^59^. It is therefore possible that cAMP-independent Epac1 accumulation at the PM reduces interaction of Epac1 with microtubules and thereby impairs microtubule dynamics involved in neurite formation. Alternatively, it is possible that PM Epac1 induces changes in the expression or function of receptors that regulate cellular adhesion, including integrins^21^. Notably, this study showed that Epac1 expression increases cell adhesion, even without addition of an Epac1 agonist. This can be due to endogenous cAMP, but may also point towards a cAMP-independent effect of Epac1 on integrins. In the context of our findings it is also of interest that integrins contribute to regulation of neurite outgrowth in different neuronal cell types^60^,^61^,^62^,^63^. Further studies will be needed to understand how cAMP-independent Epac1 PM accumulation inhibits neurite outgrowth.

The question arises whether *in vivo* situations exist in which impβ1 levels are low enough to lead to Epac1 PM accumulation and subsequent inhibition of neuronal outgrowth. Interestingly, while impβ1 protein is readily detectable in the neuronal cell bodies in dorsal root ganglia, the protein is not detectable in sciatic nerve axons^42^,^46^. Epac1 is likely present in the peripheral axon because intraplantar 8-pCPT injection rapidly induces a pain response that is prevented in Epac1 knockout mice^64^. Interestingly, axonal levels of impβ1 rapidly increase by local synthesis of the protein in response to neuronal injury^42^,^46^. Axonal impβ1 protein synthesis is dependent on a specific impβ1 mRNA transcript that has an axonal localization signal in its 3′ UTR. Specific deletion of this axon-targeted impβ1 mRNA prevents the injury-induced increase in axonal impβ1 protein and inhibits recovery in mice with sciatic nerve injury. The authors ascribed the reduced recovery to deficient retrograde injury signaling^46^. On the basis of our findings, we predict that under basal conditions, the low level or even absence of axonal impβ1 allows Epac1 to be present at the PM and to inhibit neuronal outgrowth. In situations of nerve damage, impβ1 will be produced locally in the axons, which will prevent Epac1 PM accumulation and thereby preclude inhibition of neurite outgrowth and repair.

## Materials and Methods

### Materials

High glucose Dulbecco’s modified Eagle medium (DMEM) was purchased from GE Healthcare (Piscataway, NJ, USA), fetal bovine serum (FBS) and TrypLE-express from Gibco (Carlsbad, CA, USA). Live cell imaging solution was purchased from Life Technologies (Eugene, OR, USA). Glass bottom μ-dishes for live cell imaging were from ibidi (Madison, WI, USA). 8-(4-Chlorophenylthio)-2′-O-methyladenosine-3′,5′-cyclic monophosphate acetoxymethyl ester (8-pCPT-AM) was purchased from BioLog Life Science Institute (Bremen, Germany). The primary antibodies: Epac1 (for WB), importin β1, and Rap1 were from Cell Signaling (Danvers, MA, USA); Epac1 A-5 (for IP of endogenous Epac1) and H-70 (for immunogold labeling) were from Santa Cruz (Dallas, TX, USA), GFP from Clontech (Mountain View, CA, USA); βIII-tubulin from Abcam (Cambridge, MA, USA). The HRP-conjugated secondary antibodies were from Jackson ImmunoResearch Laboratories (West Grove, PA, USA), Alexa Fluor 594 goat anti-rabbit was from Invitrogen (Carlsbad, CA, USA). FluorSave™ Reagent was from Calbiochem (Temecula, CA, USA).The chemiluminescence detection reagent was purchased from GE Healthcare Life Sciences (Pittsburgh, PA, USA). The transfection reagents jetPRIME^®^ was from Polyplud transfection (New York, NY, USA), TransIT-Neural^®^,and TransIT^®^-LT1 were from Mirus (Madison, WI, USA). GFP-Trap^®^_A beads were from Allele Biotechnology (San Diego, CA, USA) and Protein G-sepharose and protein A-sepharose were from GE Healthcare Life Sciences (Pittsburgh, PA, USA). Ral GDS-RBD precoupled agarose beads (Millipore, Temecula, CA, USA). Membrane Lipid Arrays were from Echelon Biosciences (Salt Lake City, UT, USA). All other reagents were from (Sigma-Aldrich, St. Louis, MO, USA) unless specified otherwise.

All Epac1 mutant constructs were generated using either GENEART^®^ Site-Directed Mutagenesis System from Invitrogen (Carlsbad, CA, USA) or Q5^®^ Site-Directed Mutagenesis Kit from NEB and validated by DNA sequencing. Importin β1 si-RNA (FlexiTube Mm_Kpnb1_5) and (FlexiTube Hs_KPNB1_1), and control si-RNA (AllStars Neg. Control) were purchased from Qiagen (Cambridge, MA, USA).

### Cell culture and transfection

HEK293, N2A, and EA.hy926 cells were grown in 5% CO2 at 37 °C in DMEM containing 10% FBS, 100 U/ml penicillin, and 100 μg/ml streptomycin. When seeding cells for experiments, plates were coated with poly-L-lysine to aid attachment. Transfections of si-RNA and Epac1 plasmids were performed using jetPrime for HEK293 and EA.hy926 cells or TransIT-Neural or LT1 for N2A cells, according to the manufacturer's instructions. Overexpression or knockdown were verified by western blotting.

### Pulldown assay

Cells were lysed in buffer containing 10 mM Tris-HCl pH 7.5, 150 mM NaCl, 1 mM EDTA, 0.5% NP-40, 10 mM NaF, 5 mM β-glycero-phosphate, 1 mM Na_3_VO_4_, and protease inhibitor cocktail, for 30 min at 4 °C. Lysates were clarified by centrifugation at 20,000 × g for 10 min at 4 °C. For pull-down of YFP-Epac1, equal amounts of protein lysate were incubated with GFP-Trap^®^_A beads for 2 hrs, washed three times and eluted with SDS-sample buffer. For immunoprecipitation of impβ1, lysates pre-cleared with IgG were incubated with 1 μg of impβ1 antibody for 1 hr at 4 °C, followed by addition of 50% slurry of protein G-sepharose and further incubation for 2 hrs at 4 °C. For immunoprecipitation of endogenous Epac1, lysates from four 10 cm plates per condition were pre-cleared with IgG then incubated with 1 μg of Epac1 antibody (A-5) overnight, followed by addition of 50% slurry of protein A-sepharose and further incubation for 2 hrs at 4 °C. The precipitate was washed three times and eluted with SDS sample buffer. Proteins were separated by SDS–PAGE and analyzed by western blotting. Signal was detected using the GE LAS 4000 imager (GE Healthcare, Marlborough, MA, USA).

For mass spectrometry analyses, proteins from the pulldown complex were resolved by SDS-PAGE and silver stained using the SilverQuest™ Silver Staining Kit (Invitrogen, Carlsbad, CA, USA) according to manufacturer’s instructions. Bands between 72 and 120 kDa were excised digested with trypsin, and analyzed by high-sensitivity LC-MS/MS on an Orbitrap Elite mass spectrometer (Thermo Scientific, San Jose, CA, USA). Spectra were searched against the Swiss-Prot database (EBI) using Mascot (v2.3, Matrix Science, London, UK).

### Microscopy and immunostaining

N2A cells overexpressing GFP, WT or mutant YFP-Epac1 were treated with si-impβ1 or si-ctl and incubated with either regular medium (RM) or serum-free medium (SFM) for 24 hrs. Live cell images were acquired using a SPE Leica Confocal Microscope (Leica Microsystems, Buffalo Grove, IL, USA) with a 63X objective, and analyzed with LAS X software.

For immunostaining, cells were fixed with 4% formaldehyde in PBS, treated with 0.25% Triton X-100, blocked in 2% BSA/PBS and stained with anti-βIII-tubulin antibody in blocking buffer followed by AlexaFluor 594 goat and DAPI. Images were acquired using a SPE Leica Confocal Microscope with a 63X objective.

For assessment of neurite length, bright field, and YFP images were acquired using an EVOS^®^ FL Auto Imaging System (Life Technologies, Grand Island, USA) with a 10X objective. Neurite lengths in cells expressing the fluorescent construct were measured using the EVOS^®^ FL Auto Software.

### Immuno-electron microscopy

The electron microscopy (EM) method for visualizing inner PM leaflet localized proteins has been previously published^44^,^45^. In brief, EA.hy926 cells grown on glass coverslips were washed with PBS and blotted using filter paper. Copper EM grids were placed on top of cells with pioloform- and poly-L-lysine-coated surface facing the cells and pressure applied with a rubber bung to attach the grids to the cells. Surface pressure of a 10 μl KoAc bubble placed onto the glass coverslip floats off the EM grids with sheets of apical PM attached away from the coverslip. PM sheets were then fixed using 4% paraformaldehyde and 0.1% glutaraldehyde. Endogenous Epac on PM sheets was immunolabeled with 4.5 nm gold nanoparticles coupled directly to Epac1 antibody (H-70). Gold distribution was visualized using transmission EM. The number of gold particles within an area of 1 μm^2^ of an intact PM sheet was counted using ImageJ. At least 15 PM sheets were imaged per condition.

### FRET analyses by Flow cytometry

Flow FRET assays of cells transfected with the FRET probe ECFP-Epac1-citrine were performed using an LSR Fortessa X-20 Analyzer (BD Biosciences). Cells were excited with the 488 nm laser and YFP emission was collected with a 530/30 filter. For FRET analysis, citrine was excited with the 405 nm laser and signal was measured with the 530/30 filter. For each sample > 30,000 YFP positive cells were analyzed using FlowJo software (Tree Star Inc. Ashland, OR). FRET measurements are given as a percent of vehicle-treated cells in si-ctl samples.

### Rap1-GTP pull-down assay

Cells were treated with 0.1 μM 8-pCPT-AM or vehicle for 15 min at 37 °C and lyzed in ice-cold buffer containing 50 mM Tris, pH 7.5, 200 mM NaCl, 2 mM MgCl_2_, 1% NP-40, 10% glycerol, 10 mM NaF, 5 mM β-glycero-phosphate, 1 mM Na_3_VO_4_ and protease inhibitor cocktail. Lysates were clarified by centrifugation at 14,000 × g for 10 min at 4 °C. To pull down Rap1-GTP, equal amounts of total protein were incubated with Ral-GDS beads for 1.5 hr at 4 °C with rotating followed by centrifugation at 14,000 × g for 1 min. Washed beads were suspended in 1X SDS-sample buffer. Samples were resolved by SDS-PAGE and analyzed by western blotting.

### Protein/Lipid overlay assay

Membranes spotted with a concentration gradient (1.56–100 pmol/spot) of phospholipids, were incubated in blocking buffer containing 3% BSA in 50 mM Tris-HCl, pH 7.5, 150 mM NaCl, 0.1% Tween20 for 1 hr at room temperature as previously described^34^. They were then incubated with 0.5 μg/ml of purified GST-Epac1 in the absence or presence of 50 μM 8-pCPT for 15 min at room temperature. Membranes were probed with the Epac1 antibody. DAG: diacylglycerol, PA: phosphatidic acid, PS: phosphatidylserine, PI: phosphatidylinositol, PE: phosphatidylethanolamine, PC: phosphatidylcholine, PG: phosphatidylglycerol, SM: sphingomyelin.

### Statistical analyses

Data are expressed as mean ± SEM of three or more independent experiments. Statistical analyses were performed using two-way analysis of variance (ANOVA) followed by Tukey’s multiple comparisons test or using Student’s t-test as appropriate.

## Additional Information

**How to cite this article**: Baameur, F. *et al.* Epac1 interacts with importin β1 and controls neurite outgrowth independently of cAMP and Rap1. *Sci. Rep.* **6**, 36370; doi: 10.1038/srep36370 (2016).

**Publisher’s note**: Springer Nature remains neutral with regard to jurisdictional claims in published maps and institutional affiliations.

## Supplementary Material

Supplementary Information

## Figures and Tables

**Table 1:** Mass spectrometry analyses.

Protein	Mass (Da)	Score (Matches)
Impβ1	97108	301 (10)
RanGAP1	63502	120 (3)
HSP70-1A	70009	599 (20)
HSP71	70854	175 (11)
HSP90α	84607	147 (5)
HSP90β	83212	136 (6)
HSP70-2	69978	61 (2)

Score corresponds to the number of all observed spectra of matching peptides (shown between parentheses) for a given protein, the highest the score the most confident match. Identified proteins containing at least two matching peptides are listed.
