# Supplementary material for: Epac1 interacts with importin β1 and controls neurite outgrowth independently of cAMP and Rap1
Source: Sci Rep. 2016 Nov 3;6:36370. doi: 10.1038/srep36370 (PMC5093460; doi:10.1038/srep36370)
Supplement: Supplementary Information [file srep36370-s1.doc]

**Supplementary Information**

**Epac1 interacts with importin β1 and controls neurite outgrowth independently of cAMP and Rap1**

Faiza Baameur^1^, Pooja Singhmar^1^, Yong Zhou^2^, John F. Hancock^2^, Xiaodong Cheng^2^, Cobi J. Heijnen^1^, Annemieke Kavelaars^1^

**Methods:**

**DNA-PK assay**

Cells were treated with 1 μM 8-pCPT-AM or vehicle for 15 min at 37°C and homogenized in ice-cold buffer containing 70 mM sucrose, 210 mM mannitol, 5 mM Hepes, pH 7.9, 1 mM EDTA, and protease inhibitor cocktail. Nuclear fractions were isolated as previously described ^1^. Samples were resolved by SDS-PAGE and analyzed by western blotting.

**Supplementary Figure 1**

**
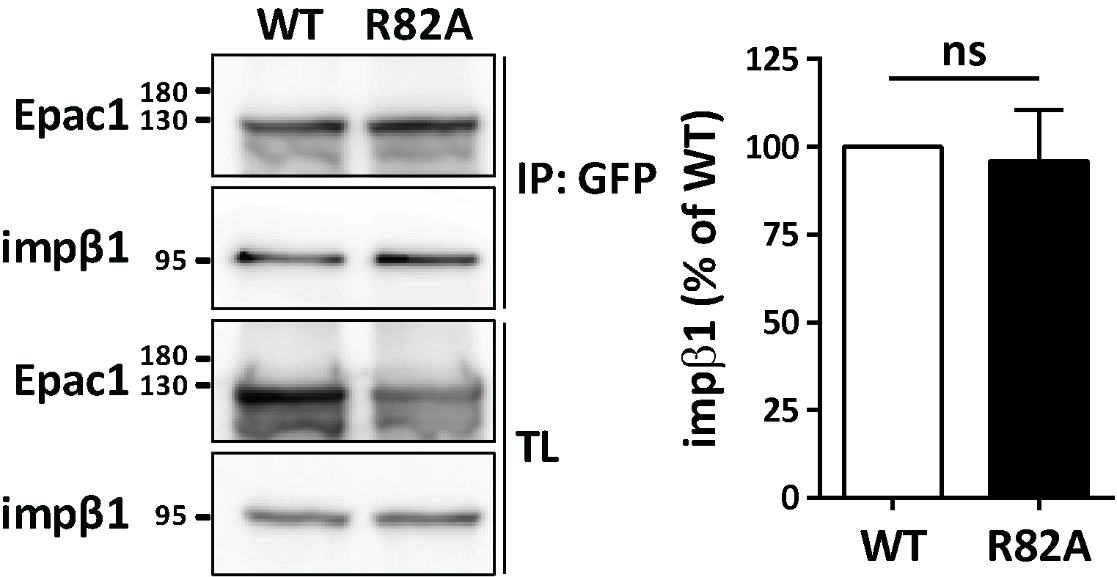
**

**Figure S1:** GFP-TRAP precipitates from cells expressing YFP-Epac1 or YFP-Epac-R82A were analyzed by western blotting with impβ1 and Epac1 antibodies. Levels of impβ1 and Epac1 in the immunoprecipitates (IP) and total lysate (TL) are shown in representative western blots. Quantification of the results of four independent experiments of the immunoprecipitated impβ1 normalized to the level of immunoprecipitated Epac1 is shown in the right panel.

**Supplementary Figure 2**

**
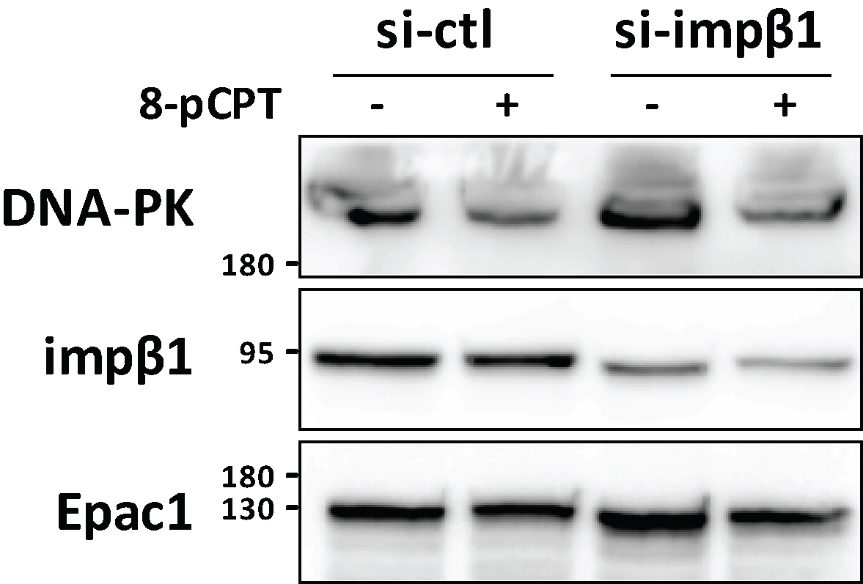
**

**Figure S2:** Cells overexpressing YFP-Epac1 were treated with si-ctl or si-impβ1, followed by treatment with vehicle or 1 µM 8-pCPT for 15 min. Nuclear fractions were isolated and analyzed for DNA-PK by western blot. As a control total Epac1 and impβ1 are shown.

**References:**

1 Nijboer, C. *H. et a*l. Gender-specific neuroprotection by 2-iminobiotin after hypoxia-ischemia in the neonatal rat via a nitric oxide independent pathway*. J Cereb Blood Flow Met*a**b** 27, 282-292, doi:10.1038/sj.jcbfm.9600342 (2007).
